# Discovery of potent and highly selective sodium-dependent glucose cotransporter 2 (SGLT2) inhibitors for treating diabetic nephropathy

**DOI:** 10.3389/fphar.2026.1776423

**Published:** 2026-04-08

**Authors:** Haotian Ni, Yifei Geng, Shan Xu, Yuting Wang, Dong Sun, Fengzhen Wang

**Affiliations:** 1 Department of Pharmacy, The Affiliated Hospital of Xuzhou Medical University, Xuzhou, Jiangsu, China; 2 Department of Pharmaceutical Analysis, China Pharmaceutical University, Nanjing, Jiangsu, China; 3 Clinical Research Center For Kidney Disease, Xuzhou Medical University, Xuzhou, Jiangsu, China

**Keywords:** biological evaluation, diabetic nephropathy, pharmacophore modeling, sodium-glucose cotransporter 2, virtual screening

## Abstract

Inhibition of glucose reabsorption by SGLT2 in the kidney is a promising strategy for the treatment of diabetic nephropathy. Adverse side effects of SGLT1 inhibition can be reduced by selective inhibition of SGLT2. Here, we developed an integrated virtual screening approach (combining drug-likeness analysis, pharmacophore model recognition, molecular docking experiments, and interaction analysis) to discover novel SGLT2 inhibitors. *In vitro* experiments suggested that all of the hits had strong inhibitory effects against SGLT2 with IC_50_ values ranging from 1.5 nM to 196 nM. Particularly, hit 4 showed an excellent inhibition of SGLT2 (IC_50_ = 1.5 ± 0.3 nM) and high SGLT1/SGLT2 selectivity of 3510-fold. Furthermore, *in vivo* treatment experiments showed it could enhance the renal glucose excretion, reduce the blood glucose and ameliorate renal function in the diabetic mice. Our results indicate that hit 4 is expected to become a promising drug candidate for treating diabetic nephropathy.

## 1 Introduction

The number of people with diabetic nephropathy is increased by 74% in recent 20 years worldwide and is likely to increase sharply in the coming decades (Ricciardi and Gnudi, 2021). Recently, inhibition of glucose reabsorption in the kidneys has become a viable strategy for ameliorating renal function in patients with diabetic nephropathy (Ravindran and Munusamy, 2022; Shaffner et al., 2021). More than 99% of the blood glucose filtered in healthy glomerulus is reabsorbed by the proximal tubules to maintain glucose homeostasis (Asano, 2003; Kinne and Castaneda, 2011; Mende, 2022). Sodium-glucose co-transporters (SGLTs) mediate glucose reabsorption in the human kidney. Under normal physiological conditions, SGLT2 is a low-affinity and high-capacity cotransporter responsible for about 90% of renal glucose reabsorption, while the remaining 10% of glucose is reabsorbed by the high-affinity and low-capacity transporter protein SGLT1 (Kakinuma et al., 2010; Mackenzie et al., 1996; Nomura, 2010). Furthermore, in experiments measuring 24-h glucose excretion in knockout mice, it was observed that all filtered glucose was excreted in the urine in the dual SGLT1/SGLT2 knockout model, while renal glucose reabsorption capacity was reduced to 67% and 98% in single-knockout SGLT2 and SGLT1 mice, respectively. In addition, recent studies indicate that SGLT2-deficient individuals only have significant renal glycosuria (up to 140 g per day) without other adverse effects (Chavda et al., 2021). On the other hand, in contrast to SGLT2, expressed almost exclusively in the kidney, SGLT1 not only serves as a glucose transporter in the renal tubules, but is also abundantly expressed in the small intestine (Bhatt et al., 2021). Evidence indicate that SGLT1-deficient individuals appear to delay glucose absorption and reduce postprandial glucose. However, the diarrhoea may be caused by inhibition of intestinal SGLT1 (Bhatt et al., 2021; Tuttle et al., 2021; Wright, 2021). Therefore, we focus on the strategy of highly selective inhibition of SGLT2 for the treatment of patients with diabetic nephropathy in this study.

The first SGLT inhibitor studied was the natural product phlorizin that decreased the level of plasma glucose in diabetic rodents (Martín et al., 1996). But as it is a dual inhibitor of both SGLT1 and SGLT2, it was found to cause severe side effects in the gastrointestinal tract. In addition, considering the inherent metabolic instability of phlorizin to β-glucosidase, clinical investigators considered that relatively high doses would be required to exert the expected efficacy (Turk et al., 1991). Therefore, phlorizin is not an ideal drug for clinical use. T-1095 was the first reported structural derivative of phlorizin, followed by sergliflozin and remogliflozin, and all are representatives of O-glucosides that act as SGLT2 inhibitors (Connelly et al., 2018; Hu et al., 2018; Rossetti et al., 1987). However, because of metabolic instability, poor therapeutic efficacy *in vivo*, and severe side-effects of O-glucoside SGLT2 inhibitors, the research of these compounds has been discontinued in preclinical trials or clinical trials (Cao et al., 2016; Dobbins et al., 2012). Although several C-glucoside SGLT2 inhibitors, such as dapagliflozin and empagliflozin, have been approved and demonstrated remarkable cardio-renal protective effects, their selectivity profiles over SGLT1 vary significantly. Current clinical debates suggest that while partial SGLT1 inhibition might supplement glucose lowering, it simultaneously increases the risk of gastrointestinal adverse events, such as osmotic diarrhea, which may complicate the treatment of frail patients with advanced diabetic nephropathy (Mudaliar et al., 2015; Spatola et al., 2018). Furthermore, the precise renoprotective mechanisms beyond systemic glucose lowering remain a subject of intense investigation (Chen et al., 2023; Dai et al., 2023). Therefore, discovering novel scaffolds with superior SGLT2 selectivity is essential not only to minimize SGLT1-mediated side effects but also to provide more precise chemical tools for decoupling the complex physiological interplay between these two transporters in the progression of diabetic nephropathy. Consequently, the development of potent and highly selective SGLT2 inhibitors is urgently required.

Previous studies used structure-based virtual screening methods to successfully identify a series of lead compounds targeting therapeutic targets from databases (Ehrenkranz et al., 2005; Lim, 2019; Liu et al., 2020; Zhou et al., 2019a). Notably, an integrated strategy combining *in silico* screening and experimental validation has also been widely applied in the discovery of anti-diabetic agents targeting other pathways, which further demonstrates the reliability of this methodological framework in anti-diabetic drug research (Dong et al., 2024). Here, to explore potent and highly selective SGLT2 inhibitors, we constructed an integrated screening approach combining drug-likeness analysis, pharmacophore recognition, virtual screening, molecular docking, and interaction analysis. Based on this approach, four-hit compounds were finally identified as potential inhibitors with nanomolar inhibitory activities against SGLT2. Among them, hit 4 was the most active SGLT2 inhibitor and exhibited very high selectivity for SGLT2 with a 3510-fold ratio of IC_50_ values (SGLT1/SGLT2). This selectivity ratio is notably higher than that of the clinically approved SGLT2 inhibitor empagliflozin (1086-fold) and the natural SGLT inhibitor phlorizin (4-fold), which better illustrates the significance of its subtype selectivity over SGLT1. Most importantly, hit 4 could enhance the renal glucose excretion, reduce the blood glucose and ameliorate renal function in the diabetic mice, indicating the potential of treating diabetic nephropathy *in vivo*.

## 2 Materials and methods

### 2.1 The establishment and validation of pharmacophore model

The high-resolution crystal structure of SGLT was retrieved in the Protein Data Bank (PDB ID: 3DH4). Firstly, the protein structure was prepared using the QuickPrep function of Molecular Operating Environment (MOE) software (Liu et al., 2020; Vilar et al., 2008), including protonated structures, removal of unbound water, and energy minimization [force field: OPLS-AA force field]. Secondly, the binding site of the original ligand (galactose) was used to determine the binding pocket of the protein target. Ligand Interaction tool was applied to identify the galactose-protein interaction. According to the interaction between galactose and SGLT active site amino acids, pharmacophore features were created at the binding site using Pharmacophore Query Editor. These pharmacophores represent the key features required for ligand binding.

Using the GH scoring method to determine the accuracy and selectivity of the model, and determine the ability of recall actives from a test data set that includes both known actives and inactives (Liu et al., 2020; Lu et al., 2011). This test dataset was constructed by combining 25 known SGLT2 active compounds and 1975 inactive decoy molecules retrieved from the Directory of Useful Decoys-Enhanced (DUD-E) database—a widely accepted standard for virtual screening validation (Mysinger et al., 2012). Pharmacophore Search Protocol of MOE was used to screen the testing dataset and assess the hit lists by the following formula:

$$GH=\left(\frac{Ha\left(3A+Ht\right)}{4HtA}\right)\left(1-\frac{\left(Ht-Ha\right)}{\left(D-A\right)}\right)$$

Statistical parameters were calculated including total molecules in the database (D), total hits (Ht), active hits (Ha), and goodness of hit score (GH) were calculated.

### 2.2 Virtual screening

The virtual screening in this study was performed by MOE software. An in-house database of 35,000 compounds was used in this work, as originally established and reported in our previous study (Zhou et al., 2019b). This database primarily consists of synthetic compounds accumulated by our research group and collaborative studies, supplemented with a small number of commercially available, novel-scaffold compounds. It covers diverse structural types, including heterocycles, aromatic systems, aliphatic derivatives, and natural product-like molecules, showing good structural diversity and broad chemical space representation. Additionally, several representative known SGLT2 inhibitors were included as positive controls to validate the screening system and ensure the accuracy of subsequent activity evaluation. The energy minimization procedure of MOE was used to convert the 2D chemical structures into 3D structures [gradient: 0.05, force field: MMFF94]. First, to improve drug-likeness, all 35,000 compounds were filtered using the Lipinski’s rule criteria. According to the physicochemical properties of SGLT2 inhibitors, the drug-like hit compounds we filtered met these parameters: (i) molecular weight ≤500; (ii) hydrogen-bond donor group ≤5; (iii) hydrogen-bond acceptor group ≤10; (iv) the octanol/water partition coefficient (Log P) value ≤5. Next, based on the pharmacophore model generated above, the Pharmacophore Search Protocol in MOE was used to screen compounds from the database. The RMSD was calculated to assess the matching degree between each compound’s pharmacophore features and the model’s corresponding feature points. This is a pharmacophore feature-matching RMSD that quantifies the spatial deviation between the compound’s key pharmacophore features and the model’s feature points. A smaller RMSD indicates a higher spatial matching degree, suggesting greater potential for binding to the SGLT2 active pocket. Based on MOE’s standard screening criteria and pre-experimental validation, compounds satisfying all model features with an RMSD cutoff of ≤0.6 Å were finally selected.

### 2.3 Molecular docking experiments

The high-resolution crystal structure of SGLT was downloaded from the Protein Data Bank (PDB ID: 3DH4, resolution: 2.73 Å, from *Vibrio* parahaemolyticus). Although vSGLT is of bacterial origin, it shares a highly conserved LeuT-fold with human SGLT2 (hSGLT2). To validate the reliability of this model for human-targeted drug discovery, a structural superposition between vSGLT and the hSGLT2 model was performed. The core transmembrane domains (TM1–TM10) exhibited an excellent alignment with an RMSD of less than 0.01 Å (Supplementary Figure S1). Specifically, the residues forming the substrate-binding pocket are highly conserved in spatial orientation and chemical environment between the two species, justifying the use of vSGLT as a structural surrogate for hSGLT2 in our docking studies.

The protein structure was prepared using the preparation tool of MOE, involving protonation of the structure, removal of unbound water molecules, and energy minimization (Liu et al., 2020). The original ligand for the SGLT2 active site (galactose) was used to define the binding pocket for molecular docking. The Triangle matcher algorithm was employed for conformation search, and 30 optimal poses were generated for each small molecule for subsequent scoring and screening. To eliminate the bias of a single scoring function, a two-step scoring strategy was implemented: initial rapid screening was conducted using the London dG scoring function, and the retained favorable poses were further rescored with the independent GBVI/WSA dG scoring function for unbiased evaluation of binding affinity. After scoring, conformation clustering analysis was performed on the generated poses of each molecule based on their spatial position and orientation in the binding pocket. The pose with the largest cluster size and the optimal comprehensive score from the two scoring steps was selected as the representative conformation of each compound, and the final hit compounds were ranked by the dG scoring for subsequent interaction analysis and visualization.

### 2.4 *In vitro* SGLT2 inhibition assay

Stably transfected Chinese hamster ovary-K1 (CHO-K1) cells that expressed human SGLT2 and human SGLT1 were seeded (30,000 cells/well) into 96-well plates (Corning, NY, USA) in the Ham’s F-12 (Gibco, Grand Island, NY, USA) supplemented with 10% fetal bovine serum (FBS, Gibco, Australia) at 37 °C in a 5% CO_2_ atmosphere for 48 h. The cells were incubated in reaction buffer containing 1 mM methyl-α-D-glucopyranoside (AMG; Sigma-Aldrich, St. Louis, MO) supplemented with [^14^C] AMG and serial dilutions of the test compounds (0.001, 0.003, 0.01, 0.03, 0.1, 0.3, 1, 3, 10, 30, 100, 300, 1,000, 3,000, 10,000 nM) for 45 min at 37 °C (Ohtake et al., 2012). The test compounds included the hits 1-4 and reference standards phlorizin and empagliflozin, which were tested under identical conditions. AMG uptake activity was measured by counting the radioactivity of the cell lysates using a liquid scintillation counter (PerkinElmer, Waltham, MA, USA). IC_50_ values were calculated using GraphPad software fitted with a sigmoidal dose-response model. All experiments were performed in triplicate (n = 3), and data are presented as mean ± standard deviation (SD). Selectivity ratios were calculated by dividing the IC_50_ value for SGLT1 by the IC50 value for SGLT2 for each compound.

### 2.5 Molecular dynamics simulations

The crystal structure of the SGLT from *vibrio* parahaemolyticus (PDB ID: 3DH4) was downloaded from the PDB, and hits 1-4 were respectively docked into the binding pocket using MOE to generate the initial protein-ligand complex structures, which served as the starting coordinates for all simulations. Given that SGLT is a transmembrane protein, all simulations were conducted within an explicit phospholipid bilayer. The AMBER99SB-ILDN force field parameters for the protein were generated using the Acpype tool, while ligand topology files were derived based on the GAFF force field and AM1-BCC charge calculations. The protein-ligand complexes were embedded into a pre-equilibrated POPC phospholipid bilayer, with the orientation determined by aligning the hydrophobic region of the protein with the hydrophobic core of the bilayer. The systems were placed in a periodic box, solvated with SPC water molecules, and neutralized by adding Na^+^ and Cl^−^ ions to achieve physiological salt concentration. A 50,000-step steepest descent energy minimization was first performed to eliminate unrealistic atomic clashes. Subsequently, multi-stage equilibration was conducted: initially, a 100 ps NVT simulation with position restraints was performed using the V-rescale thermostat to couple the system temperature to 300 K; this was followed by a 100 ps NPT simulation using the Parinello-Rahman barostat to maintain the pressure at 1 bar, employing semi-isotropic pressure coupling to prevent collapse of the lipid bilayer. Following equilibration, three independent 100 ns simulations were carried out for each system, employing the LINCS algorithm to constrain bond lengths involving hydrogen atoms. Long-range electrostatic interactions were calculated using the Particle Mesh Ewald method, and the van der Waals cutoff was set to 1.0 nm. Trajectory data were saved every 10 ps. Root mean square deviation (RMSD) and the number of hydrogen bonds between protein and ligand were calculated using GROMACS built-in tools, and binding free energies were computed via the MM-PBSA method.

### 2.6 Serum stability test

Human serum was thawed at 37 °C and centrifuged at 12,000 × g for 10 min at 4 °C to remove insoluble debris. The supernatant was collected and diluted to 25% (v/v) with phosphate-buffered saline (PBS, pH 7.4). Hit 4 was added to the diluted serum to a final concentration of 5 μM, and the mixture was incubated at 37 °C with gentle shaking. At predetermined time points (0, 30, 60, 120, 240, and 360 min), 200 μL aliquots of the reaction mixture were withdrawn and immediately mixed with an equal volume (200 μL) of ice-cold acetonitrile to terminate the reaction and precipitate serum proteins. After incubation at 4 °C overnight, the mixture was centrifuged at 12,000 × g for 15 min at 4 °C, and the supernatant was filtered through a 0.22 μm membrane filter for high-performance liquid chromatography (HPLC) analysis. The residual amount of hit 4 at each time point was calculated as a percentage relative to the initial amount (0 min), based on the integral area of the characteristic HPLC peak corresponding to hit 4. The results are presented as mean ± standard deviation (SD) from three independent experiments.

### 2.7 *In vivo* assay

Male db/db mice were obtained from Vital River (Beijing, China). All animal procedures and experiments were approved by the Animal Ethics Committee of Xuzhou Medical University (NO. 20210226192). Mice were randomly assigned to vehicle and treatment groups (n = 6). Compounds were orally administered to nonfasted db/db mice at 1, 5, and 10 mg/kg. The dose selection was based on literature precedent for SGLT2 inhibitors (Ohtake et al., 2012) and adjusted to characterize the dose-response relationship of the test compounds. Blood samples were collected from the tail vein immediately before dosing at 0, 1, 2, 4, and 6 h after dosing. Urine samples were collected from metabolic cages within 6 h post-dosing. All assessments were performed by an investigator blinded to the group assignments to eliminate observer bias. Blood and urine glucose concentrations were determined by the hexokinase method, and urinary creatinine concentrations were measured by the enzymatic approach.

### 2.8 Statistical analysis

The mean ± SD was used to express all results. Statistical analyses were performed using GraphPad Prism (version 9.0; GraphPad Software, San Diego, CA, USA). Normality of data distribution was assessed using the Shapiro-Wilk test, and homogeneity of variances was evaluated using Levene’s test. For comparisons between two groups, an unpaired two-tailed Student’s t-test was used. For comparisons involving multiple groups, a one-way analysis of variance (ANOVA) was performed, followed by Dunnett’s *post hoc* test to compare each treatment group to the control group. Significance levels at *p* < 0.001 were considered statistically significant.

## 3 Results and discussion

### 3.1 The establishment and validation of pharmacophore model

**FIGURE 1:**
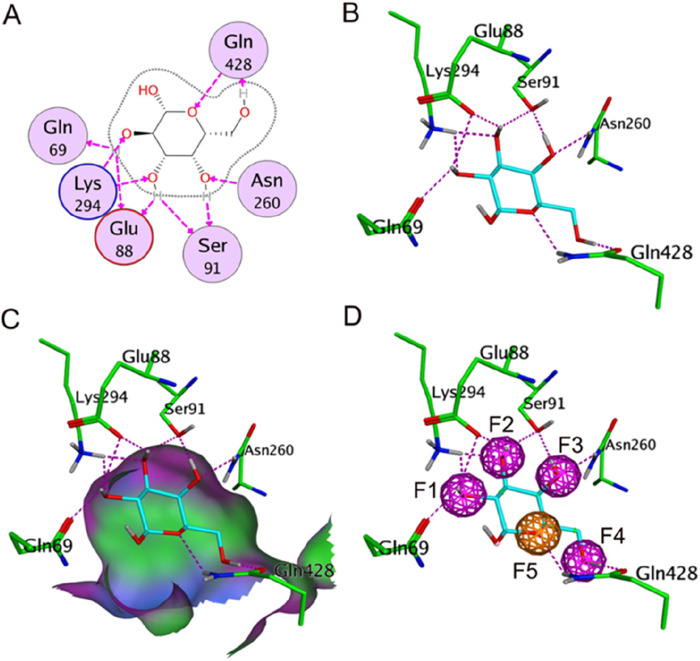
**(A)** 2D representation of the interaction of galactose with key amino-acid residues in the active site. **(B)** 3D representation of the interaction of galactose. **(C)** Molecular surface representation of the SGLT-galactose interface. **(D)** The generated structure-based pharmacophore model using MOE. Bound galactose is shown as cyan stick form; amino-acid residues are depicted by green stick form. Purple dotted lines show the hydrogen-bond interactions with the key residues. F1-F4, hydrogen-bond donor feature (purple); F5, hydrogen-bond acceptor feature (orange).

Our previous studies have fully confirmed that receptor-ligand pharmacophore generation is an efficient and reliable alternative to identify potential inhibitors (Ehrenkranz et al., 2005; Lim, 2019; Liu et al., 2020; Zhou et al., 2019c). It models receptor-ligand interaction considering the critical contact points in the binding site. In this research, the crystal structure of the SGLT-ligand complex (PDB ID: 3DH4, Resolution: 2.73 Å) was used as a reference to establish the pharmacophore model. The interactions of the binding ligand galactose with active pocket protein residues were shown in Figures 1A–C, five oxygen atoms of the galactose formed multiple hydrogen bonds with the key residues, including Lys294, Glu88, Gln69, Ser91, Gln428, and Asn260. To understand the comprehensive information on galactose binding, a structure-based pharmacophore model comprising four hydrogen-bond donor features (F1-F4) and one hydrogen-bond acceptor feature (F5) was constructed based on the interaction relationship of protein-galactose. The generated pharmacophore and the spatial arrangement of the model, as well as the chemical interactions are depicted in Figure 1D. Four hydroxyl groups of galactose engaged in hydrogen-bond interactions with the active-site residues (Lys294, Glu88, Gln69, Ser91, Gln428, and Asn260) mapped the four F1-F4 features of the pharmacophore model. In addition, the oxygen atom of the galactose that interacts with Gln428 by hydrogen bond corresponds to the F5 feature. These mapping results of the galactose pharmacophore features to the model indicated its potential identification of active compounds.

The pharmacophore model was further validated by the GH score method (Liu et al., 2020). The virtual screening of a testing dataset containing 25 active SGLT2 inhibitors (collected from the reported literature (Zhou et al., 2021; Zhou et al., 2019b)) and 1975 inactive compounds was searched by the Pharmacophore Search Protocol in MOE. The pharmacophore model retrieved 28 molecules with 23 active inhibitors. A series of statistical parameters, consisting of the total hits (*Ht*), active hits (*Ha*), false negatives, false positives, enrichment factor (*E*), and goodness of hit score (*GH*), were calculated (Table 1). The *GH* score method was used to determine the selectivity of the model and select out the actives from the decoy set. A *GH* score should be greater than 0.7, which indicates a reliable model (0 indicates the null model, 1 indicates the ideal model). The GH score of 0.84 indicated the good quality of the pharmacophore model based on the validated data.

**TABLE 1:** Pharmacophore model validation using the *GH* score method.

Serial no.	Parameter	Pharmacophore model
1	Total molecules in the database (*D*)	2000
2	Total number of actives in the database (*A*)	25
3	Total hits (*Ht*)	28
4	Active hits (*Ha*)	23
5	% Yield of actives [(*Ha*/*Ht*) × 100]	82%
6	% Ratio of actives [(*Ha*/*A*) × 100]	92%
7	Enrichment factor (*E*) [(*Ha* × *D*)/(*Ht* × *A*)]	66
8	False negatives [*A*-*Ha*]	2
9	False positives [*Ht*-*Ha*]	5
10	The goodness of hit score (*GH*)	0.84

### 3.2 Virtual screening and molecular docking

**FIGURE 2:**
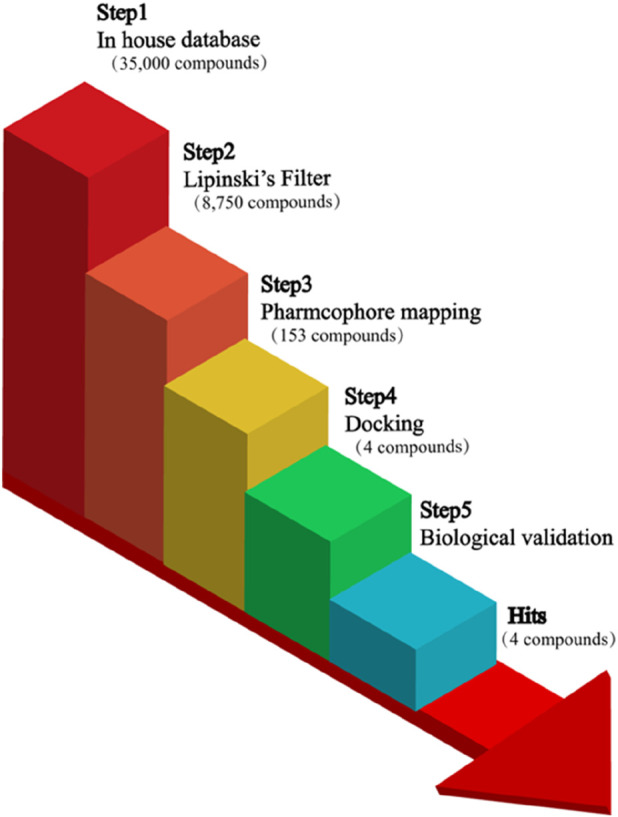
The virtual screening workflow represented hit compounds obtained by drug-likeness analysis, pharmacophore-based screening, molecular docking, and biological evaluation.

A hierarchical virtual screening was carried out using drug-likeness analysis, pharmacophore recognition, molecular docking tests, and lastly visual examination of the binding modes of the hit compounds. As shown in Figure 2, 35,000 compounds of the in-house database were firstly filtered to satisfy drug-like properties by applying Lipinski’s rule of five as a filtration protocol to end up with 8,750 hit compounds (Liu et al., 2020). Then, we applied the validated 3D pharmacophore model as an input search query to select 153 hit compounds with an RMSD value of less than 0.6 Å from 8,750 drug-like compounds with the Pharmacophore Search Protocol. These retrieved hits that survived the filtration steps were then followed by molecular docking.

**FIGURE 3:**
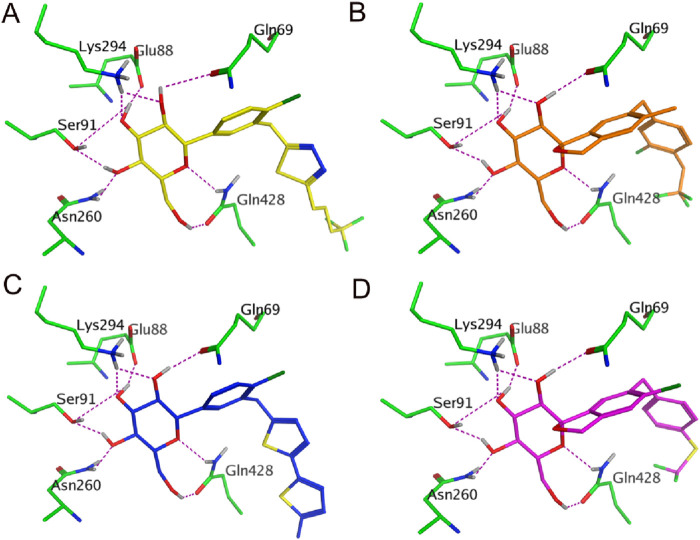
3D representation of the interactions of hit compounds with the key amino-acid residues. **(A)** Hit 1 (yellow). **(B)** Hit 2 (orange). **(C)** Hit 3 (blue). **(D)** Hit 4 (purple). Amino acids are depicted by green stick form; purple dotted lines indicate the hydrogen-bond interactions with residues.

**FIGURE 4:**
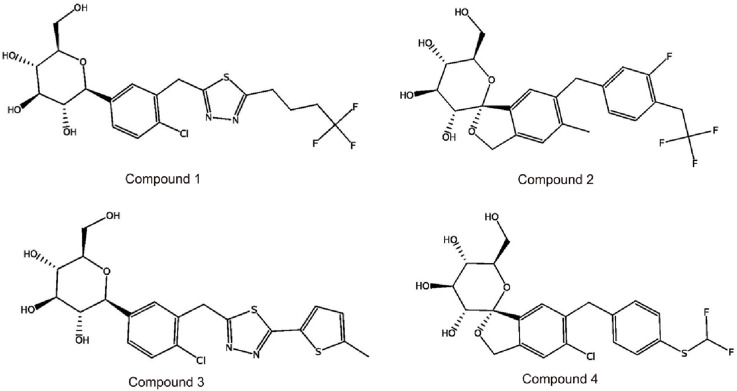
The chemical structures of four hit compounds (Hit 1–4).

**FIGURE 5:**
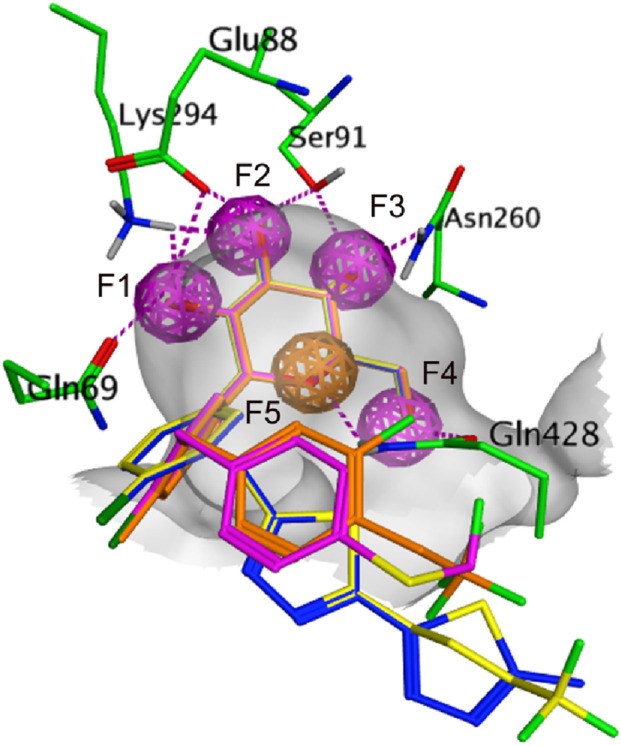
The mapping of hits 1-4 with pharmacophore features. Hit compounds and residues are rendered as sticks. F1-F4, hydrogen-bond donor feature (purple); F5, hydrogen-bond acceptor feature (orange).

Subsequently, these hit compounds were screened by docking with the active site of SGLT2. To prioritize the most promising candidates with the highest predicted binding affinities, we analyzed the score distribution of the 153 retrieved hits (Supplementary Table S1). According to the docking score distribution, we selected the four hit compounds (termed as hits 1–4) with exceptionally low docking scores below −15 kcal/mol, representing the top-tier candidates of the screened library. By exploiting the structural information of their binding modes with active-site residues, we visually examined the docked poses of the four hits. As shown in Figures 3, 4, all four hits exhibited multiple hydrogen-bond interactions with the key residues (Lys294, Glu88, Gln69, Ser91, Gln428, and Asn260). Moreover, these hits perfectly mapped the five pharmacophore features (Figure 5). Finally, the four compounds were selected with good drug-like properties, the lowest RMSD values, and good docking scores for *in vitro* and *in vivo* testing.

### 3.3 *In vitro* SGLT2 inhibition assay

We further evaluated the ability of the hits 1-4 to inhibit SGLT2 *in vitro* and their selectivity for SGLT1 inhibition. Hit 1 selectively inhibits SGLT2 approximately three times as much as SGLT1 inhibition and showed moderate inhibitory activity (IC_50_ = 196 ± 32 nM for SGLT2) (Table 2). Compared with hit 1, hit 2-4 showed more potent inhibitory effects against SGLT2, and their IC_50_ values are between 1 and 20 nM. Hit 2 showed a potent inhibition activity toward SGLT2 (IC_50_ = 6 ± 1.2 nM) and was about 25 times more selective for SGLT2 than for SGLT1. Hit 4 strongly inhibited SGLT2 activity (IC_50_ = 1.5 ± 0.3 nM), indicating that hit 4 is about ten and two times more potent than phlorizin and empagliflozin, respectively. Besides, the SGLT1 inhibitory activity of hit 4 was 5,265 ± 340 nM, and its selectivity for SGLT2 was 3510-fold, more significant than the selectivity of phlorizin and empagliflozin. Hits 2 and 4, which exhibited potent SGLT2 inhibitory activity were nominated for further evaluation *in vivo*.

**TABLE 2:** Drug-like properties, RMSD values, docking scores, and biological activities of hit compounds.

Hits	MW^a^	HBD^b^	HBA^c^	LogP^d^	RMSD [Å]^e^	Docking score [kcal/mol]	IC_50_ [nM]^f^		
							SGLT2	SGLT1	Selectivity^g^
1	483	4	7	2.8	0.5596	−15.1345	196 ± 32	618 ± 52	3
2	472	4	6	3.5	0.5579	−15.9764	6 ± 1.2	152 ± 34	25
3	469	4	7	2.6	0.5587	−15.6038	18.3 ± 0.5	879 ± 210	48
4	475	4	6	3.8	0.5575	−16.3803	1.5 ± 0.3	5,265 ± 340	3,510
Phlorizin	​	​	​	​	​	​	18 ± 0.6	69 ± 21	4
Empagliflozin	​	​	​	​	​	​	3.1 ± 0.9	3,367 ± 262	1,086

^a^ Molecular weight.

^b^ Number of hydrogen bond donor atoms.

^c^ Number of hydrogen bond acceptor atoms.

^d^ Log of the octanol/water partition coefficient.

^e^ Root of the mean square distance (RMSD) of pharmacophore with the mapping ligands (lower values of RMSD, means better pharmacophore mapping with ligands).

^f^ IC_50_ values are represented as mean ± SD (n = 3).

^g^ The selectivity values were calculated by IC_50_ SGLT1/IC_50_ SGLT2.

### 3.4 Molecular dynamics simulations

**FIGURE 6:**
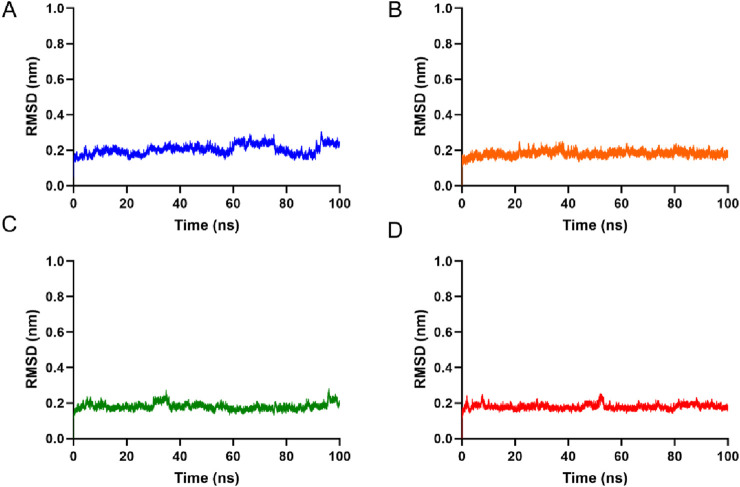
Root-mean-square deviation (RMSD) of (hits 1–4) in complexes over 100 ns MD simulations: **(A)** hit 1, **(B)** hit 2, **(C)** hit 3, **(D)** hit 4. All systems were simulated in triplicate.

**FIGURE 7:**
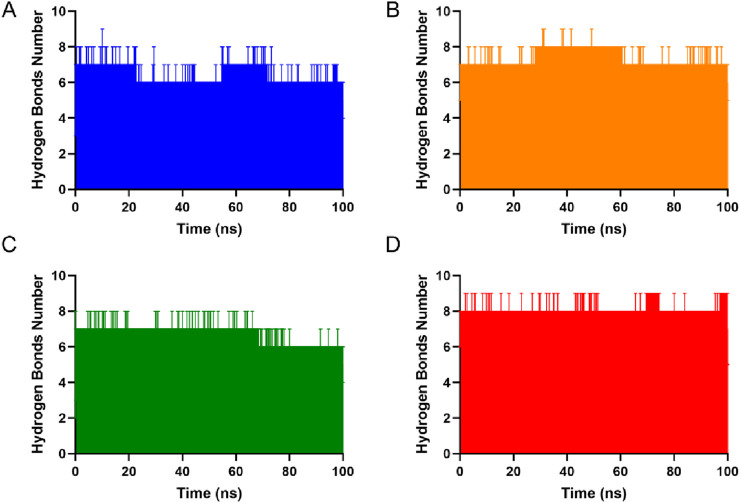
Hydrogen bond counts of hits 1-4 **(A–D)** in complexes over a 100 ns molecular dynamics simulation period. All simulations were performed in triplicate.

To evaluate the binding stability of hits 1-4 with SGLT, three independent 100 ns molecular dynamics simulations were performed for each system. Analysis of the root mean square deviation (RMSD) of the protein backbone atoms relative to the initial structure revealed that all four complexes maintained stable conformations throughout the simulations. As shown in Figure 6, the RMSD values for each replicate exhibited minor fluctuations during the initial 10 ns equilibration period and subsequently stabilized within a narrow range of approximately 0.2–0.3 nm for the remainder of the 100 ns trajectory, with no significant drift or abrupt conformational transitions observed across the triplicate runs. This consistent behavior indicates robust structural integrity of the complexes over the simulated timescale. The stability of the protein-ligand interactions was further assessed by monitoring hydrogen bonds formed between each compound and SGLT. As shown in Figure 7, the number of hydrogen bonds remained relatively stable throughout the 100 ns simulations for all systems. Notably, hit 4 maintained the most consistent hydrogen bond interactions, with only minor fluctuations observed over the entire trajectory. The persistent hydrogen bonding networks, coupled with the stable RMSD profiles, confirm that hydrogen bonds serve as the predominant non-covalent interactions anchoring the compounds within the SGLT2 binding pocket. Quantitative assessment of binding free energies using the MM-PBSA method revealed highly favorable interactions for all four compounds, with total binding free energies ranging from −2083.74 to −2,276.50 kcal/mol (Supplementary Table S2). The rank order of binding affinity was hit 4 > hit 2 > hit 3 > hit 1, with hit-4 exhibiting the most negative total binding free energy (−2,276.50 ± 82.19 kcal/mol), suggesting the strongest binding affinity among the series. Decomposition of the free energy terms revealed that electrostatic interactions constituted the predominant contribution to total binding energy (−6136.76 to −6296.90 kcal/mol), consistent with the hydrogen bond analysis demonstrating stable polar interactions. Notably, hit 4 displayed the most favorable electrostatic energy (−6296.90 ± 105.22 kcal/mol), further supporting the formation of an optimally stabilized hydrogen bonding network with SGLT. The synergistic combination of favorable van der Waals interactions and solvation energies for hit 4 contributes to its thermodynamically optimal binding profile, providing a structural and energetic basis for its potential as an SGLT inhibitor candidate. Collectively, these findings demonstrate that all four compounds form stable complexes with SGLT, with hit 4 exhibiting the most favorable binding characteristics.

### 3.5 Serum stability of hit 4

**FIGURE 8:**
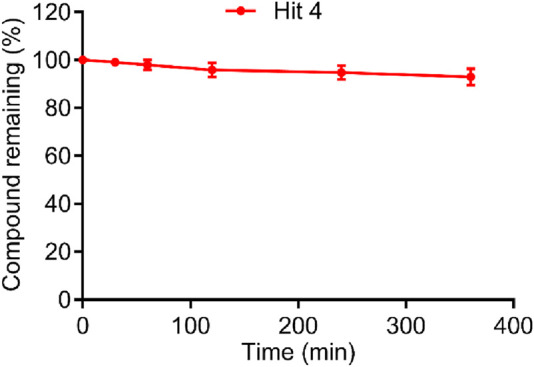
The biostability of hit 4 in human serum. Data are shown as mean ± SD (n = 3).

To evaluate the *in vitro* biostability of the most promising candidate hit 4, we performed serum stability assay. The results showed that hit 4 remained highly stable in human serum during the entire incubation period, with a residual rate of more than 85% at 6 h post-incubation (Figure 8). This excellent serum stability of hit 4 indicated that it could resist degradation by serum enzymes and maintain its structural integrity in the circulatory system, which provides a critical pharmacokinetic basis for its effective *in vivo* efficacy and supports its potential as a clinical drug candidate for diabetic nephropathy. As a key ADMET property, this metabolic stability lays a solid experimental foundation for preclinical development of hit 4, and comprehensive characterization of its full ADMET profile will be explored in subsequent studies.

### 3.6 *In vivo* efficacy in diabetic db/db mice

**FIGURE 9:**
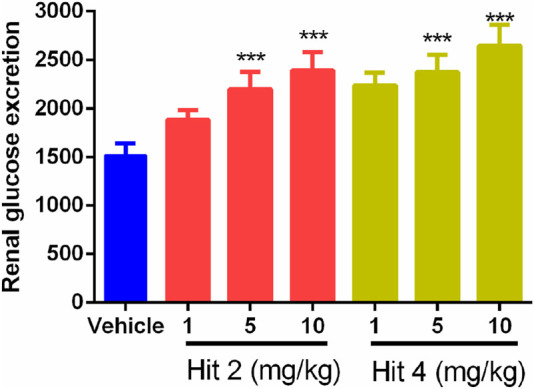
The effect of single oral administration of hit 2 and hit 4 on renal glucose excretion in db/db mice (0–6 h). Renal glucose excretion (urine glucose concentration/urine creatinine concentration) was calculated from the urine collected at 0–6 h after administration under nonfasting conditions. ***p < 0.001 versus vehicle group. Data are represented as mean ± SD (n = 6).

**FIGURE 10:**
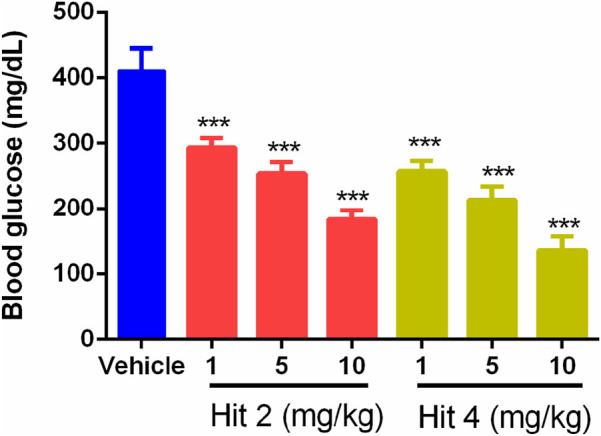
The effect of single oral administration of hit 2 and hit 4 on blood glucose levels in db/db mice at 6 h after administration. ***p < 0.001 versus vehicle group. Data are represented as mean ± SD (n = 6).

Based on the excellent *in vitro* SGLT2 inhibition of hit 2 and hit 4, the *in vivo* effects of hit 2 and hit 4 on diabetic mice were further evaluated. The effects of hit 2 and hit 4 on renal glucose excretion and blood glucose levels in db/db mice were evaluated at three rational oral doses (1 mg/kg, 5 mg/kg and 10 mg/kg). After oral administration of hit 2 and hit 4 at different doses, the results in Figure 9 showed that the renal glucose excretion was significantly increased within 0–6 h. Furthermore, these two hits reduced the blood glucose level to the normal range in a dose-dependent manner, but the blood glucose concentration was not less than 100 mg/dL (Figure 10), suggesting that they could improve hyperglycemia and ameliorate renal function without causing hypoglycemia. Therefore, hit 2 and hit 4 were considered effective drugs for treating diabetic nephropathy.

## 4 Conclusion

This study has constructed an integrated computational approach combining drug-likeness analysis, pharmacophore recognition, virtual screening, molecular docking, and interaction analysis with key amino acids and successfully identified four potent SGLT2 inhibitors with nanomolar inhibitory activity. Remarkably, the most active inhibitor hit 4 showed more potent inhibitory activity (IC_50_ = 1.5 ± 0.3 nM) and higher selectivity for SGLT2 with a 3510-fold ratio of IC_50_ values (SGLT1/SGLT2) than phlorizin, indicating that the integrated approach is a feasible strategy for the discovery of potent SGLT2 inhibitors. Most importantly, hit 4 could improve renal glucose excretion, reduce the blood glucose and ameliorate renal function in diabetic db/db mice. Notably, hit 4 also showed excellent serum metabolic stability, a key ADMET property that consolidates its preclinical development potential. Future studies will conduct comprehensive ADMET characterization to further support its clinical translation for diabetic nephropathy treatment. Furthermore, the integrated screening approach developed herein may also provide guidance for mining molecular databases to discover more potent and selective SGLT2 inhibitors.

## Data Availability

The original contributions presented in the study are included in the article/Supplementary Material, further inquiries can be directed to the corresponding authors.
