# Supplementary material for: Discovery of potent and highly selective sodium-dependent glucose cotransporter 2 (SGLT2) inhibitors for treating diabetic nephropathy
Source: Front Pharmacol. 2026 Apr 8;17:1776423. doi: 10.3389/fphar.2026.1776423 (PMC13099297; doi:10.3389/fphar.2026.1776423)
Supplement: Supplementary file 1 [file Supplementaryfile1.docx]

***Supplementary Material***

**Discovery of potent and highly selective sodium-dependent glucose cotransporter 2 (SGLT2) inhibitors for treating diabetic nephropathy**

**Haotian Ni^†,1^, Yifei Geng^†,2^, Shan Xu^†,3^,** **Yuting Wang^2^, Dong Sun^*,3^, Fengzhen Wang^*,1,3^**

***Correspondence:** **Fengzhen Wang, xydwfzh@xzhmu.edu.cn**


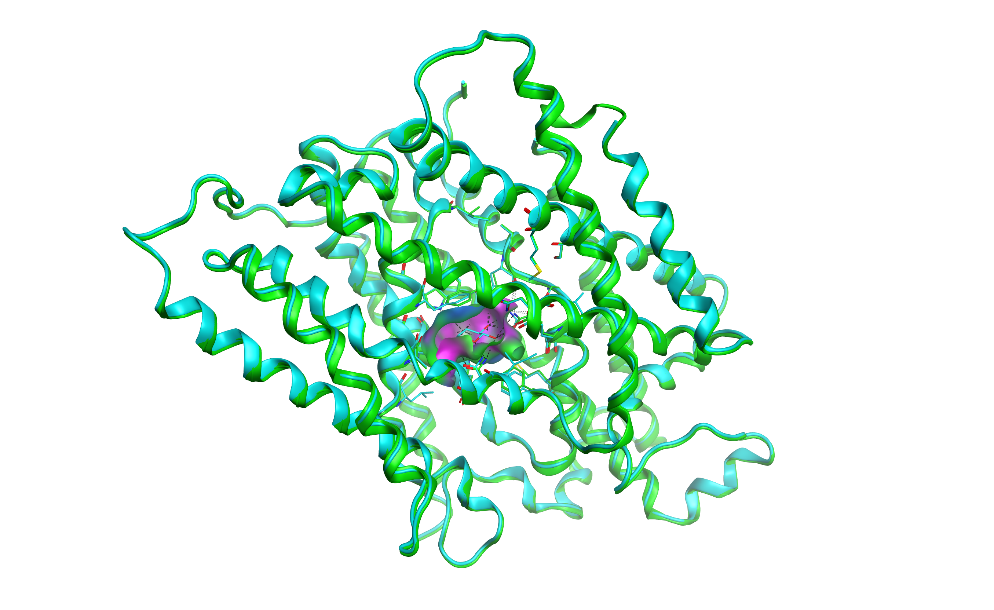


**Figure S1.** Structural superposition of the bacterial SGLT (cyan, PDB ID: 3DH4) and the human SGLT2 model (green, PDB ID: 8HIN). The alignment demonstrates high structural conservation within the core transmembrane domains and the substrate-binding pocket (RMSD < 0.01 Å).

**Table S1.** Docking scores of the 153 candidate compounds identified from the pharmacophore-based virtual screening.

| **Hits** | **Docking score [kcal/mol]** |
| --- | --- |
| 1 | –15.1345 |
| 2 | –15.9764 |
| 3 | –15.6038 |
| 4 | –16.3803 |
| 5 | -14.7557 |
| 6 | -14.7313 |
| 7 | -14.4979 |
| 8 | -14.3812 |
| 9 | -14.3706 |
| 10 | -14.2312 |
| 11 | -14.1998 |
| 12 | -14.121 |
| 13 | -14.0789 |
| 14 | -14.0414 |
| 15 | -14.0166 |
| 16 | -13.9207 |
| 17 | -13.7812 |
| 18 | -13.6302 |
| 19 | -13.5592 |
| 20 | -13.5181 |
| 21 | -13.5082 |
| 22 | -13.5008 |
| 23 | -13.4417 |
| 24 | -13.4249 |
| 25 | -13.3222 |
| 26 | -13.2549 |
| 27 | -13.2372 |
| 28 | -13.2286 |
| 29 | -13.1891 |
| 30 | -13.1641 |
| 31 | -13.1551 |
| 32 | -13.1429 |
| 33 | -13.1217 |
| 34 | -13.1201 |
| 35 | -13.1099 |
| 36 | -13.0953 |
| 37 | -13.0103 |
| 38 | -12.9515 |
| 39 | -12.9505 |
| 40 | -12.9447 |
| 41 | -12.9217 |
| 42 | -12.9098 |
| 43 | -12.903 |
| 44 | -12.8601 |
| 45 | -12.8095 |
| 46 | -12.7991 |
| 47 | -12.7961 |
| 48 | -12.7555 |
| 49 | -12.7479 |
| 50 | -12.7294 |
| 51 | -12.717 |
| 52 | -12.6908 |
| 53 | -12.6888 |
| 54 | -12.6769 |
| 55 | -12.6755 |
| 56 | -12.6335 |
| 57 | -12.6205 |
| 58 | -12.0259 |
| 59 | -12.0154 |
| 60 | -12.0117 |
| 61 | -12.0083 |
| 62 | -12.005 |
| 63 | -11.9938 |
| 64 | -11.9812 |
| 65 | -11.9651 |
| 66 | -11.9498 |
| 67 | -11.9498 |
| 68 | -11.9368 |
| 69 | -11.9294 |
| 70 | -11.9277 |
| 71 | -11.8873 |
| 72 | -11.8503 |
| 73 | -11.8455 |
| 74 | -11.8314 |
| 75 | -11.8186 |
| 76 | -11.7964 |
| 77 | -11.7926 |
| 78 | -11.7814 |
| 79 | -11.7686 |
| 80 | -11.7578 |
| 81 | -11.7546 |
| 82 | -11.7536 |
| 83 | -11.7485 |
| 84 | -11.7351 |
| 85 | -11.7343 |
| 86 | -11.7057 |
| 87 | -11.7015 |
| 88 | -11.6912 |
| 89 | -11.6881 |
| 90 | -11.6879 |
| 91 | -10.9942 |
| 92 | -10.985 |
| 93 | -10.9768 |
| 94 | -10.9753 |
| 95 | -10.9751 |
| 96 | -10.9642 |
| 97 | -10.9563 |
| 98 | -10.9528 |
| 99 | -10.9449 |
| 100 | -10.941 |
| 101 | -10.9396 |
| 102 | -10.9175 |
| 103 | -10.9159 |
| 104 | -10.9118 |
| 105 | -10.9097 |
| 106 | -10.906 |
| 107 | -10.9008 |
| 108 | -10.8968 |
| 109 | -10.8917 |
| 110 | -10.8887 |
| 111 | -10.887 |
| 112 | -10.8728 |
| 113 | -10.8723 |
| 114 | -10.8715 |
| 115 | -10.867 |
| 116 | -10.8614 |
| 117 | -10.8598 |
| 118 | -10.077 |
| 119 | -10.074 |
| 120 | -10.0706 |
| 121 | -10.0618 |
| 122 | -10.0418 |
| 123 | -10.0328 |
| 124 | -10.0187 |
| 125 | -10.0168 |
| 126 | -10.0068 |
| 127 | -10.0015 |
| 128 | -9.9936 |
| 129 | -9.9768 |
| 130 | -9.9702 |
| 131 | -9.9616 |
| 132 | -9.9593 |
| 133 | -9.9584 |
| 134 | -9.9534 |
| 135 | -9.9301 |
| 136 | -9.9202 |
| 137 | -9.9118 |
| 138 | -9.9086 |
| 139 | -9.906 |
| 140 | -9.8934 |
| 141 | -9.8918 |
| 142 | -9.8898 |
| 143 | -9.8763 |
| 144 | -9.8728 |
| 145 | -9.8558 |
| 146 | -9.8536 |
| 147 | -9.8489 |
| 148 | -9.8307 |
| 149 | -9.8285 |
| 150 | -9.8253 |
| 151 | -9.8181 |
| 152 | -9.6492 |
| 153 | -9.3402 |

**Table S2.** MM-PBSA binding free energies (kcal/mol) of the hits 1-4 in complex over a 100 ns simulation. Data are shown as mean ± SD (n = 3).

| **Energy terms (kcal/ mol)** | **hit-1** | **hit-2** | **hit-3** | **hit-4** |
| --- | --- | --- | --- | --- |
| van der Waals energy | -684.79 ± 24.78 | -698.58 ± 19.64 | -692.26 ± 30.18 | -721.95 ± 27.47 |
| electrostatic energy | -6136.76 ±143.96 | -6219.11 ± 116.33 | -6200.11 ± 128.92 | -6296.90 ± 105.22 |
| polar solvation energy | -1187.67 ± 44.52 | -1324.07 ± 53.14 | -1215.41 ± 50.65 | -1337.28 ± 48.26 |
| nonpolar solvation energy | 41.64 ± 3.57 | 50.93 ± 4.17 | 47.27 ± 3.38 | 58.40 ± 5.06 |
| total gas-phase free energy | -837.71 ± 52.16 | -911.31 ± 47.31 | -888.95 ± 43.83 | -930.63 ± 51.17 |
| total solvation free energy | -1146.03 ± 38.49 | -1242.13 ± 36.26 | -1192.14 ± 42.17 | -1295.88 ± 40.94 |
| total binding free energy | -2083.74 ± 93.04 | -2193.45 ± 87.28 | -2131.09 ± 78.32 | -2276.50 ± 82.19 |
